# Promoting Healthy Food Access and Nutrition in Primary Care: A Systematic Scoping Review of Food Prescription Programs

**DOI:** 10.1177/08901171211056584

**Published:** 2021-12-10

**Authors:** Matthew Little, Ebony Rosa, Cole Heasley, Aiza Asif, Warren Dodd, Abby Richter

**Affiliations:** 1 School of Public Health and Social Policy, 8205 University of Victoria, Victoria, BC, Canada; 2 Department of Population Medicine, University of Guelph, Guelph, ON, Canada; 3 Department of Chemistry, 3653 University of Guelph, Guelph, ON, Canada; 4 Department of Biomedical Sciences, 3653 University of Guelph, Guelph, ON, Canada; 5 School of Public Health Sciences, 8430 University of Waterloo, Waterloo, ON, Canada; 6 Guelph Community Health Center, Guelph, ON, Canada

**Keywords:** systematic scoping review, food prescriptions, social prescribing, food security, food is medicine, nutrition, health promotion, population health

## Abstract

**Objective:**

To conduct a scoping review to synthesize evidence on food prescription programs.

**Data Source:**

A systematic search of PubMed, CINAHL, Web of Science, Embase, and the Cochrane Library was conducted using key words related to setting, interventions, and outcomes.

**Study Inclusion and Exclusion Criteria:**

Publications were eligible if they reported food prescription administered by a health care practitioner (HCP) with the explicit aim of improving healthy food access and consumption, food security (FS), or health.

**Data Extraction:**

A data charting form was used to extract relevant details on intervention characteristics, study methodology, and key findings.

**Data Synthesis:**

Study and intervention characteristics were summarized. We undertook a thematic analysis to identify and report on themes. A critical appraisal of study quality was conducted using the Mixed Methods Appraisal Tool (MMAT).

**Results:**

A total of 6145 abstracts were screened and 23 manuscripts were included in the review. Food prescriptions may improve fruit and vegetable consumption and reduce food insecurity (FI). Evidence for impacts on diet-related health outcomes is limited and mixed. The overall quality of included studies was weak. Addressing barriers such as stigma, transportation, and poor nutrition literacy may increase utilization of food prescriptions.

**Conclusion:**

Food prescriptions are a promising health care intervention. There is a need for rigorous studies that incorporate larger sample sizes, control groups, and validated assessments of dietary intake, food security, and health.

## Introduction

According to the Global Burden of Disease Study (GBD) 2016, suboptimal diet is the second-leading risk factor for deaths and disability-adjusted life years (DALYs) globally, accounting for 18.8% of all deaths and almost 10% of all DALYs.^1^ It is well-established that poor diets (e.g., a diet low in whole grains, fruit, and vegetables and high in sodium, refined grains, and sugar) are associated with higher risk of chronic diseases, including cardiovascular disease and resulting mortality.^2^ Dietary behaviors and consumption are shaped by inter-related personal and environmental factors, including education and knowledge,^3,4^ prices and affordability,^5^ physical environments and accessibility,^6^ marketing and regulations,^7,8^ and vendor and product properties.^9^ Public health and health care models emphasizing social determinants of health have contributed to the growing recognition that one’s social, cultural, economic, and geographical positions play central roles in the accessibility and affordability of healthy foods.^10^ Food insecurity (FI) and diet-related diseases have thus been recognized as health inequities emerging from social, economic, and political structures.^11^ It is now widely recognized that in many high-income countries, healthy diets are more expensive and less accessible to some populations, including low-income and racialized communities, leading to disproportionate burdens of diet-related chronic disease among these groups.^12,13^

Recognizing the important role of social and physical environments in diet-related health, it is necessary to develop and evaluate innovative interventions that improve the accessibility, affordability, convenience, and desirability of safe and healthy foods, including whole grains, fruits, and vegetables. Building off of social prescribing models in the United Kingdom, health care practitioners (HCPs) and public health advocates are increasingly acknowledging the potential of the health care system to help patients access and consume healthy foods.^14,15^ “Food is medicine” approaches are rapidly gaining interest in North America and can be defined as interventions that subsidize or provide healthy foods to patients as a health care intervention to improve diet-related health outcomes.^15^ Embedded within this approach is the idea that individual interactions with the health care system are opportunities to offer evidence-based food and nutrition interventions to improve health outcomes and reduce health care usage and costs. Within this field of research, an area for exploration and innovation is food prescriptions. Food prescription programs generally target patients experiencing FI who are at risk of diet-related illnesses. Food prescriptions aim to improve the accessibility, affordability, and knowledge of healthy foods while reducing burdens on health care systems and reliance on medical interventions.^16^ Although intervention models vary, they often involve partnerships with food retailers (e.g., grocery store chains and farmers’ markets [FM]) to subsidize healthy foods (frequently fruits and vegetables [F&V]). A notable potential benefit over emergency food provision (e.g., food banks) and food relief programs (e.g., Supplemental Nutrition Assistance Program (SNAP) in the United States and the Farmers’ Market Nutrition Coupon Program in British Columbia, Canada) is that prescriptions are administered by HCPs, thus legitimizing the incentive while providing practitioners a pragmatic way to improve accessibility and affordability of healthy foods for their patients. Food prescription approaches also align with recent calls for improved health care-based interventions that address underlying social determinants of health and achieve improvements in health equity.^17,18^

Food prescription programs have been rapidly popularized and expanded in recent years, with several academic publications documenting the results of program evaluations and efficacy studies. However, there has been little effort to undertake a systematic synthesis to characterize and describe this literature. Considering this gap, we used a systematic scoping review methodology to synthesize available published evidence on food prescription programs with three primary objectives: (1) to characterize the aims and structures of food prescription programs; (2) to determine the effectiveness of such initiatives to improve food security (FS), food literacy, healthy food consumption, and diet-related health; and (3) to identify factors leading to the success and/or failure of such initiatives that may be relevant for practitioners, researchers, and policymakers looking to implement food prescription initiatives.

## Methods

We conducted a scoping review due to the suitability of this approach to synthesize an interdisciplinary body of literature including studies using different methodologies.^19^ Our methodology was based on frameworks published by Arksey and O’Malley^19^ and Levac and colleagues^20^ and consisted of the following five steps: (1) identifying the research questions; (2) identifying relevant studies; (3) selecting studies; (4) charting data; and (5) collating and reporting results. Methods and findings were reported according to the Preferred Reporting Items for Systematic Reviews and Meta-Analyses extension for Scoping Review checklist.^21,22^

### Search Strategy

**Figure 1.**
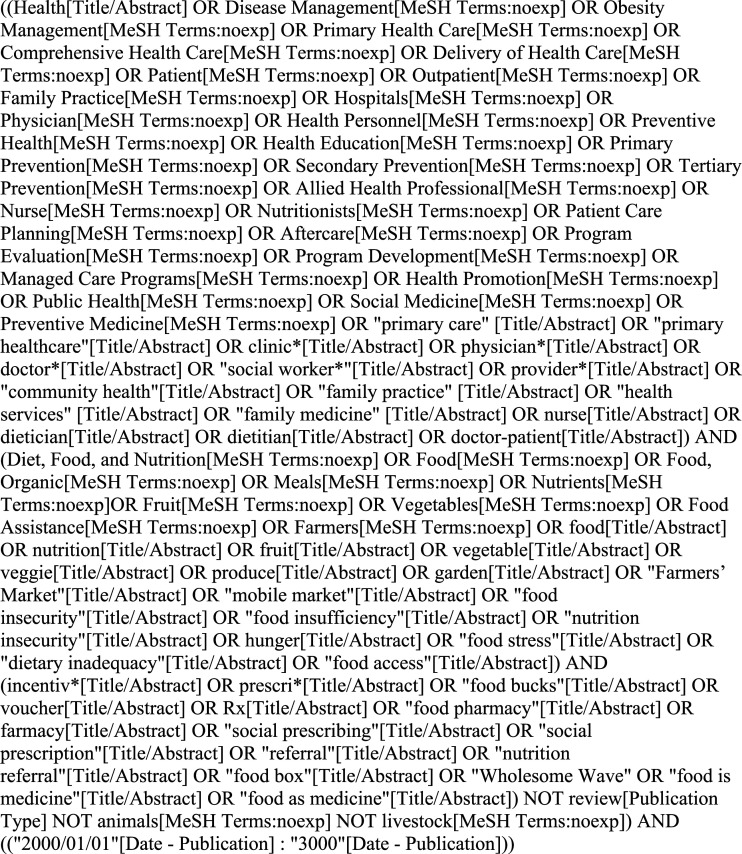
Example search strategy formatted for PubMed (Medline).

The scope and search strategy were developed in collaboration with a research librarian, HCPs at a Community Health Center, and content experts. Guided by the research team, two reviewers established the search and screening protocol a priori. A systematic search was conducted using MeSH terms and text terms in PubMed (Medline), CINAHL with Full Text (Ebsco), Web of Science (Web of Knowledge), Embase, and the Cochrane Library. Search terms included key words related to setting, interventions, program evaluations, process evaluations, and outcomes (see Figure 1). The search was limited to articles published between January 1, 2000 and April 31, 2021 to reflect that food prescriptions are a recent health care intervention. The search was conducted with the assistance of a research librarian and search terms were adapted to each database. The search included all original study types except commentaries, editorials, and systematic reviews or meta-analyses.

### Eligibility Criteria and Screening

Predetermined inclusion and exclusion criteria were used to identify eligible studies for the review. The population and location were not restricted and included people of any age group and gender from any country. Publications were eligible if they reported on interventions framed as a food, produce, or fruit and vegetable prescription administered by a HCP, including allied professionals (physicians, nurses, social workers, community health worker, dietitian, nutritionist, or midwife) with the explicit aim of improving healthy food access and consumption, FS, and/or patient health. In order to maintain an emphasis on interventions issued directly to patients within health care settings, we excluded studies in which dietary interventions and/or food vouchers issued by individuals not linked to a health care facility (e.g., academic researchers or non-medical community organizations), even if participants were recruited from a primary care facility (PCF). Studies were limited to peer-reviewed academic literature with English language abstracts.

Titles and abstracts were imported into DistillerSR (Evidence Partners), a web-based systematic review platform. Duplicates were removed using the deduplication function. Article screening was undertaken in two stages. First, titles and abstracts were screened for eligibility by two independent reviewers (E. R. and M. L.) using an eligibility form (Level 1 screening). The kappa for title and abstract screening was .84, indicating a strong level of agreement between reviewers.^23^ Second, full-text articles were screened to confirm their inclusion in the final review (Level 2 screening). The kappa score for full-text screening was .94, indicating an almost perfect level of agreement between reviewers.^23^ Independent reviewers met throughout the screening process to resolve conflicts. All articles that advanced through full-text screening were hand-searched for relevant titles within reference lists that were not captured in the initial search. Articles selected during the hand-search process underwent Level 1 and Level 2 screening. All relevant full-text articles proceeded to data extraction and analysis.

### Data Extraction and Charting

Following the screening process, full-text articles were imported to NVivo 12 (QSR International). A data charting form was developed in Microsoft Excel, which included relevant details on publication characteristics, study location, the intervention, study design and methodology, and key findings. Due to the multiple methodologies employed by studies, including arts-based and qualitative methods, we undertook a thematic analysis to identify and report on themes in the full-text articles.^19,20,24^ Thematic analysis is often used for qualitative data sets; however, its usefulness in scoping reviews has been recently recognized.^20^ The primary aim of the thematic analysis was to achieve objective (3), to identify factors leading to the success and/or failure of food prescription programs to achieve their desired impacts. A deductive-inductive approach was used to code (identify basic elements or segments of information) each full-text article by two reviewers working independently.^25^ Both reviewers worked together to merge codes into themes through an iterative process that grouped codes based on similarities and depth of supporting data. As a final step, we reviewed, defined, and named themes.^24^

### Critical Appraisal of Included Studies

Critical appraisal of study quality was conducted using the Mixed Methods Appraisal Tool (MMAT), a reliable and efficient instrument which is suitable for appraising quantitative, qualitative, and mixed methods research across multiple disciplines^26-28^ The MMAT allows for quality assessment by applying a different set of five criteria to diverse study designs, including qualitative, randomized controlled trials, non-randomized quantitative, observational descriptive, and mixed methods.^26^ Criteria act as a checklist, resulting in a quality score with a minimum of zero and a maximum of five. Quality appraisal was conducted by two reviewers working independently, who then met to discuss any conflicts and reach a consensus.

## Results

**Figure 2.**
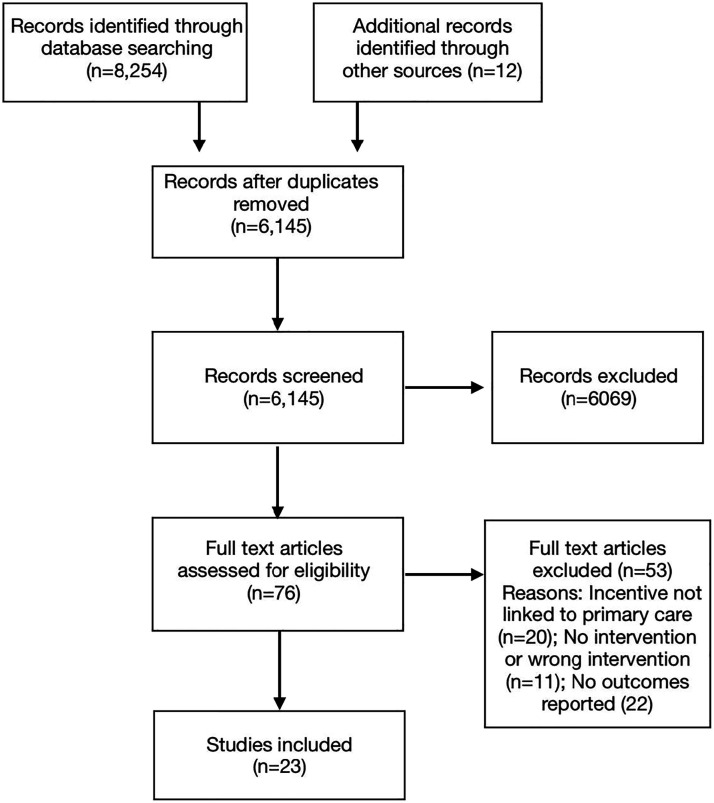
Preferred reporting items for systematic reviews and meta-analyses (PRISMA) reporting flow diagram^22^ showing four-stage article selection process used to identify articles on food prescription programs in health care settings.

Figure 2 presents the search and inclusion diagram. The literature search identified 6145 articles after deduplication. A total of 6069 articles were removed during Level 1 screening. Of the 76 full-text articles assessed for eligibility during Level 2 screening, 23 manuscripts reporting on 21 different interventions met the inclusion criteria and were included in the scoping review (Table 1). Of these, 22 manuscripts were published since 2015, indicating that food prescription programs are a new and growing area of interest for researchers. Almost all studies were conducted in the United States,^29-50^ with only one study conducted elsewhere (the United Kingdom).^51^ Most articles (n = 11) reported on a single arm (one group) repeated or pre-post measures study design with no control group,^29,32,37,40,43,45-47,49-51^ six reported on various types of program evaluations (including mixed methods feasibility, process, or outcome evaluations),^34,35,38,42,47,48^ six employed qualitative methods (including focus groups, interviews, and Photo Voice methods),^30,31,33-35,41^ one employed a retrospective case control study,^44^ and one study used a prospective cohort study.^39^

**Table 1.** Summary of Selected Studies, Including Location, Objectives, Study Design, Methodology, Data Analysis, Key Findings, and Mixed Methods Appraisal Tool quality score.

First Author (year)	Location of Intervention	Aim/Purpose	Recruitment, Participants, and Setting	Intervention/Incentive and Duration	Design of Study or Evaluation	Data Collection and Tools	Data Analysis And/or Theoretical Approach (if Relevant)	Key Findings	MMAT Quality Score
Ridberg et al., (2019)^29^	9 clinical sites across USA	To assess changes in household FS associated with a pediatric F&V Rx program	548 children and youth (2–18 years) who were clinically obese or overweight from low-income families	Physicians administered Rx at each clinical visit; Rx consisted of vouchers for fruits and vegetables worth US $0.50 to $1 per person per household per day redeemable at participating farmers' markets. Intervention lasted 4–6 months.	One group pre-post measurements	Surveys administered to parents or caregivers by HCP or staff members. FS assessed using adapted USDA 18-item household food security survey.	Paired t-tests and McNemar paired tests to compare FS outcomes between first and last clinical visit. Linear regression models were used to evaluate FS change as a function of clinical visits and voucher redemption over the course of the program.	72% of households increased FS score. Households experiencing very low FS went from 9% to 1%. Higher FS change scores with 5–6 clinical visits compared with 1–2 visits. Greater improvement in households where primary caregiver had higher education levels.	***
DeWit et al., (2020)^30^	Midwestern USA	To conduct a qualitative assessment of barriers and facilitators to F &V consumption among participants of a food Rx pilot program	29 English- or Spanish-speaking parents and caregivers (≥18 years) of children attending a pediatric clinic who screened positive for FI and were given a food Rx during the pilot program	US $5 Rx provided by pediatrician redeemable at a community mobile market selling low-cost fresh produce. Program operated for 6 months.	Qualitative study using focus groups	Semi-structured focus group guide	Grounded theory; themes were grouped and aligned with a social ecological framework.	Barriers to accessing markets and using vouchers included accessibility (unknown location, unavailability of non-produce items), affordability ($5 was insufficient to justify visit to market), and desirability (cultural barriers and spoilage of produce).	*****
Saxe-Custack et al., (2018)^41^	Flint, MI, USA	To explore caregiver perceptions of experiences with a F&V Rx program for low-income pediatric patients	32 English-speaking parents and caregivers of children attending a pediatric clinic whose children had received at least 1 fruit and vegetable Rx	US $10 vouchers provided by pediatrician during each visit to clinic, redeemable for fresh produce at a FM. When FM was closed, families had the option to receive a vendor-prepared bag of fresh fruits and vegetables. Duration of intervention not reported.	Qualitative study using semi-structured interviews	Semi-structured interview guide	Design and approach grounded in Social Cognitive Theory. Transcripts were coded in a multistep process guided by an inductive-deductive thematic analysis.	Caregivers expressed appreciation for the Rx program. Many indicated that Rx helped them acquire healthy food. Co-location of market at the pediatric clinic was beneficial and improved access. Implementation challenges included lack of clarity of program details.	*****
Cavanagh et al., (2016)^44^	Upstate NY, USA	To evaluate the “Veggie Rx” program effectiveness and determine if the intervention reduced patients' BMI	Patients at a primary care clinic classified as low-income with obesity, hypertension, and/or diabetes (108 enrolled; 54 in intervention group; 54 matched controls)	Nutritionists at PCF provided participants with 13 coupons, each with US $7 value, which could be redeemed once per week at a mobile produce market that traveled to inner-city neighborhoods. Study participants were part of Rx program for a minimum of 5 non-consecutive weeks.	Retrospective case control study	BMI (from EMR) and coupon redemption data	Two-sample t-test assessed whether the mean BMI values differed across groups and changes in BMI between the control and intervention group	Participants in intervention group experienced statistically significant decrease in BMI. Intervention group had a mean BMI decrease of .74 kg/m. Control group had a mean BMI increase of .35 kg/m (*P* = .02).	**
Jones et al., (2020)^45^	Navajo Nation, USA	To assess the impact of FVRx, a F&V Rx program on health behavior, BMI, and household food insecurity.	Navajo families with children (<7 years old) were recruited by HCP and/or Head Start staff) at 19 PCF (n = 243 enrolled; n = 212 completed pre-intervention measures; n = 122 completed post-intervention measures)	Rx administered by PCP and included 6 months’ worth of vouchers valued at US $per household member per day (max US $5 per day) redeemable for F&V and traditional foods. Participants attended monthly sessions where HCP delivered nutrition coaching and participated in cooking demonstrations and goal setting.	One group pre-post measurements	Surveys assessed F&V consumption (from Behavioral Risk Factor Surveillance System), PA, sleep, screen time, and FS (using USDA’s 6-item short form FS Questionnaire). Providers collected height and weight.	Paired t-tests to evaluate pre-post changes in BMI, F&V consumption, PA, sleep, and screen time. McNemar’s tests to assess changes in FS status.	F&V consumption increased from 5.2 to 6.8 servings per day (*P* < .001). Of children classified as overweight or obese pre-intervention, 38% achieved a healthy BMI z-score at program completion (*P* < .001). Proportion of households reporting adequate FS increased from 18% to 35% (*P* < .001).	**
Ridberg et al., (2019)^46^	12 clinical sites in Connecticut, Maine, Massachusetts, New Mexico, New York, Rhode Island, and Washington, DC, USA	To determine how F&V Rx program was associated with changes in F&V consumption	Children and youth (2–18 years) who were clinically obese or overweight from low-income families (n = 883)	Physicians administered Rx at each clinical visit; Rx consisted of vouchers for fruits and vegetables worth US $0.50-$1/person/household/day redeemable at FM. Intervention lasted 4–6 months. Program also provided in-clinic nutrition education and obesity treatment counseling.	One group pre-post measurements	Number of clinical visits and voucher redemption were recorded. Surveys administered to parents or caregivers by HCP or staff members. Survey assessed recent F&V consumption (adapted from the National Cancer Institute Eating at America’s Table Study Quick Food Scan).	Analyses included paired t-tests to compare changes in F&V consumption between first and last visits and multivariable linear regressions, including propensity dose-adjusted models, to determine factors associated with change in F&V consumption	Mean F&V consumption increased from first to last visit; participants reported a dose-response increase of .32 cups of F&V for each additional clinical visit. There was no significant dose relationship between F&V intake and voucher redemption.	****
Aiyer et al., (2019)^47^	TX, USA	To examine the feasibility, perceptions, and impact of a food Rx program in an area with a high rate of food insecurity	Adult (≥18 years) FI patients recruited from 3 PCFs by pediatricians to participate in the intervention study 8 staff and study participants were recruited for interviews (n = 242)	Primary care practitioner administered Rx, which included nutrition education materials and vouchers redeemable every 2 wk for 6 months for 30 lbs of fresh produce and healthy non-perishable items	One group pre-post mixed methods evaluation, including repeated survey measures + key informant interviews	Surveys at baseline and 3rd, 6th, 9th, and 12th redemption used a 2-item questionnaire for FS. Additional questions assessed perceived impacts on dietary intake, usage of foods provided, and estimated weekly savings on groceries. Program costs per participant per redemption were calculated.	Unpaired t-tests were used to determine change in prevalence of FS among participants over the duration of the intervention. Interview transcripts analyzed using a deductive concept-driven thematic content analysis.	Cost of program was $12 per family per week and participants reported $57 in savings per week. The study group experienced a 94.1% decrease in the prevalence of FI (*P* < .01). Key informant interviews highlighted the importance of training, ongoing support, and strong communication as factors for success. Participant interviews revealed perceived financial, dietary, and health benefits to participating in the program.	****
George et al., (2016)^48^	Pennsylvania, PA, USA	To evaluate the feasibility, strengths, and limitations of a F&V Rx program that included a nutritional mentoring component	Pediatricians recruited low-income families with overweight or obese children at weight-loss clinic (n = 4). Mentors recruited from class of medical students (n = 4).	Pediatrician issued F&V Rx, which included 4 vouchers each redeemable for US $50 of produce at a FM over a 2-month period. Families were paired with a mentor who implemented the healthSLAM curriculum on healthy eating and food preparation.	Mixed methods program evaluation	Pre-program surveys and structured interviews were conducted by mentors with participating families. Mentors participated in a focus group.	Descriptive statistics were used to characterize participants and program use. Thematic analysis was used for qualitative data.	On average, families spent 32 minutes at the market per visit, had expenditures of $40.68, and reported one weekly produce item going unused. Families valued on-site mentoring and mentors felt that it provided opportunities for professional development and improved self-care while also benefiting vendors.	***
York et al., (2020)^49^	Santa Barbara, CA, USA	To examine the feasibility and impact of a F&V Rx for Latino adults with T2D	Latino adults (≥18 y) with self-reported non-insulin treated T2D recruited by HCP and study investigators (n = 23 recruited; n = 21 completed program)	Participants received a weekly box of prescribed vegetables as determined by HCP for 12 wk at no cost to the participant	One group pre-post measurements	Pre- and post-intervention HbA1c, blood pressure, BMI, waist circumference, and FS using USDA questionnaire	Student’s t-tests and Wilcoxon signed rank tests were used to compare pre- and post-intervention measures	Over 12 wks, there was a significant drop in systolic (*P* = .03) and diastolic (*P* = .01) blood pressure. 14 participants lost weight (median weight loss 1.9 pounds) and WC decreased in 9 of 19 responders by a median of 1.5 inches. HbA1c was unchanged (6.7 ± 1.1% at baseline vs 7.0 ± 1.1% post-intervention).	***
Forbes et al., (2019)^50^	Hershey, PA, USA	To evaluate how participation in a food Rx and mentorship program changed behaviors and perceptions about healthy eating	Families or individuals (5–75 years) at risk of chronic illness or metabolic disease and had difficulty obtaining F&V by PCP (n = 10). Mentors recruited from class of medical students.	HCP administered Rx, which included US $40 of tokens per week for 12 weeks redeemable for fresh produce at a FM, along with an optional healthy recipe and shopping list. Student mentors accompanied families to the FM, helping them shop and answering additional questions.	One group pre-post measurements + 3 year follow-up interviews + written reflections by mentors via online survey	Pre-post surveys included F&V consumption, FS (2012 USDA Household FS Module), health behavior (CDC 2011 Behavioral Risk Factors Surveillance System Questionnaire), self-reported health (2007 Health Survey for England), nutrition behavior, and PA (International Physical Activity Questionnaire)	Descriptive statistics used to characterize participants and pre-post measures; no statistical tests used. Qualitative data analyzed using thematic analysis.	Following the intervention, F&V consumption increased, more patients expressed efforts to include produce in every meal, and more participants strongly agreed that F&V prevented chronic diseases. In interviews, participants appreciated the program’s ease of use, mentor-patient relationship, and increased access to produce. Mentors identified mutual benefits to participants and themselves, including skills for patient education. An identified weakness was the short duration of intervention.	****
Riemer et al., (2020)^31^	WA, USA	To determine perceived impacts of Complete Eats Rx, a F&V Rx program	Participants were eligible for program if they qualified for SNAP. For the study, Adult (>18 years old) participants were recruited at PCF from pool of individuals who had participated in the Complete Eats program in the past 6 months (n = 26).	HCPs administered Rx, which included US $10 vouchers redeemable for F&V at grocery chain retailer. Vouchers distributed per week or per visit to clinic depending on participating location. Intervention duration was 6 months.	Qualitative: Photo Voice	Each participant attended 3 focus groups. Between first and second meeting, participants voted on theme of the photography and subsequent discussions.	Not described	Complete Eats Rx was associated with perceived increases in FS and improved children’s behavior around food. Nutrition education was not perceived as beneficial without addressing major constraints to food access.	****
Bryce et al., (2017)^32^	Detroit, MI, USA	To determine the impacts of a F&V Rx program on HbA1c, BP, and weight in patients with uncontrolled T2D	Adult (≥17 y old) non-pregnant patients with T2D recruited by HCP if they had an elevated HbA1c (>6.5) within 3 months (n = 65)	HCPs issued Rx, which included up to US $40 ($10 per wk for up to 4 wk) to purchase fresh produce from a local FM	One group pre-post measurements	Blood pressure, weight, and HbA1C measured using clinical guidelines	Paired t-tests conducted to evaluate changes in HbA1C, weight, and systolic and diastolic BP	Following the intervention, a decrease in HbA1c (9.54% to 8.83%; *P* < .001) was observed. Weight and BP did not change significantly.	**
Coward et al., (2021)^33^	MS, USA	To examine health care providers’ attitudes towards food Rx interventions	HCPs (physicians, registered dietitians, and nurse practitioners) were recruited (n = 15)	N/A	Qualitative: Semi-structured phone interviews	Semi-structured interview guides targeted 4 themes: (1) barriers to implementation, (2) potential use, (3) routinizing on-boarding, and (4) nutrition education and advocacy	Summative inductive-deductive content analysis with simultaneous coding used to synthesize and analyze the interview transcripts	There was a lack of understanding by health care providers of what food Rx interventions were, how they were implemented, and what outcomes they were likely to influence. Evidence for 2 key recommendations: (1) development and validation of a screening tool to be used by clinicians for enrolling patients; and (2) implementation of nutrition education in PCP training and continuing education.	*****
Schlosser et al., (2019)^34^	Cuyahoga County, OH, USA	To understand how economic constraints shape participant engagement in a produce Rx program (PRxHTN) and sustainable F & V consumption change	Adult FI patients with hypertension were eligible for PRxHTN. A convenience sub-sample of patients (n = 23). HCPs (n = 5), and FM managers (n = 2) were recruited for the study.	Participating patients attended monthly visits with their provider for 3 months. Four US $10 vouchers to purchase produce at FMs were provided at each visit (12 weeks total). Participants were asked to set goals around F&V consumption and identify motivations for changing behavior.	Qualitative process evaluation: Semi-structured interviews	Semi-structured interview guides aimed to understand: (1) aspects of the program that did and did not work well; and (2) how patients interpreted and engaged with program.	Approach was informed by RE-AIM framework and a critical patient-centered approach with a focus on program adoption, implementation, and maintenance.	Economic hardship was a barrier to program participation and sustainability. Transportation issues shaped shopping patterns and limited participant ability to access FMs. Low and unstable income shaped participant shopping and eating behavior before, during, and after PRxHTN. Participants also emphasized individual-level influences like personal or perceived motivations for program participation.	***
Schlosser et al., (2019)^35^	Cuyahoga County, OH, USA	To evaluate the impacts of a produce Rx program (PRxHTN)	Adult FI patients with hypertension were eligible for PRxHTN. A convenience sub-sample of patients (n = 23), HCPs (n = 5), and FM managers (n = 2) were recruited for the study.	Participating patients attended monthly visits with their provider for 3 months. Four US $10 vouchers to purchase produce at FMs were provided at each visit (12 weeks total). Participants were asked to set goals around F&V consumption and identify motivations for changing behavior.	Qualitative process evaluation: Semi-structured interviews	Semi-structured interview guides	Thematic analysis, including deductive and emergent (inductive) themes regarding participant experiences. Authors compared patient, provider, and market manager perspectives.	4 central themes identified: (1) providers and patients reported positive interactions during program activities, but providers struggled to integrate the program into their workflow; (2) patients reported greater F&V intake and FM shopping; (3) social interactions enhanced program experience; (4) economic hardships influenced patient shopping and eating patterns	****
Trapl et al., (2017)^36^	Cuyahoga County, OH, USA	To examine the feasibility of integrating a Produce Rx Program for Pregnant Women (PRx) into HCP practices, ease of use of the PRx materials, and the use of FM by PRx participants	Pregnant women (<24 wk gestation) recruited by HCPs at 4 sites in priority neighborhoods (n = 75). HCPs recruited for structured interviews (n = 10).	HCPs issued a produce voucher valued at US $40 per month for 4 months redeemable for produce at local FMs. Participants were counseled and guided to create monthly nutrition-related implementation goals.	Mixed methods program evaluation	Pre-intervention questionnaire collected baseline F&V beliefs, habits, barriers to F&V consumption, and perceptions of FMs. Post-program questionnaires assessed impact of intervention on perceptions of F&Vs and FMs. Voucher redemption data were collected.	Bivariate analyses (e.g., chi-square test) used to assess differences among those who redeemed vouchers and those who did not. Interview transcripts were coded deductively based on broad categories of questions in the interview guide.	56% of participants redeemed at least one voucher and 95% reported that program materials were relevant and useful. Those who perceived having an FM close to where they lived were more likely to redeem vouchers (*P* = .016). Health care practitioners indicated that PRx created opportunities to talk about diet with participants, greater awareness about FMs, and new shopping habits.	**
Buyuktuncer et al., (2013)^51^	Castlefields Ward, UK	To assess the feasibility of a fruit and vegetable Rx program	HCPs recruited patients (≥17 years; non-targeted) from a primary health care setting (n = 621 received Rx; n = 124 baseline survey; n = 84 6-week survey; n = 54 follow-up survey)	Rx included 4 vouchers each worth GBP £1 off every £3 spent on F&V over 4 weeks at a retail store. Patients offered fruit during consultations and in the waiting room, where they were engaged by trained volunteers. Patients were also given promotional leaflets.	One group repeated measures	Telephone-based questionnaires assessed changes in F&V consumption using FACET (Five-a-Day Consumption Evaluation Tool). Other tools assessed F&V purchasing behavior, knowledge, and barriers to consumption.	Friedman test used to determine changes in consumption over time. Chi-squared test used to evaluate percentage change in relation to participant consumption of F&V. Friedman test used to rank importance of barriers to F&V consumption.	76.2% of participants used the Rx vouchers. No significant change in consumption or purchasing behavior observed (*P* > .05). Participants’ level of knowledge of F&V recommendations moderately increased over the short and medium term. Primary barriers to F&V consumption reported as the affordability and quality of fresh F&V.	*
Trapl et al., (2018)^37^	Cuyahoga County, OH, USA	To assess a produce Rx intervention, including program participation, nutrition counseling, F&V voucher redemption, and dietary behavior change	HCPs from 3 “safety net” clinics recruited FI patients (≥18 years) with hypertension (n = 224 baseline survey; n = 137 post-intervention survey)	Four US $10 vouchers to purchase produce at FMs were provided at each visit with HCP (12 weeks total). Participants were asked to set goals around increasing F&V consumption and identify motivations for changing behavior at each visit.	One group pre-post measurements	Pre-intervention surveys collected demographic characteristics, barriers to F&V consumption, and current food shopping habits. Post-intervention surveys assessed impact of the program. F&V voucher redemption data collected from FM.	Bivariate analyses compared completers (i.e., those with 3 visits to provider) and non-completers using chi-square tests. Changes in F&V and fast-food consumption were evaluated using paired t-tests.	86% visited an FM to use their produce vouchers, with one-third reporting it was their first FM visit ever. Median number of FM visits was 2 and median number of vouchers redeemed was 8. Among the sub-sample with follow-up survey data, significant improvement in F&V consumption was observed, as well as a decline in fast-food consumption.	**
Marcinkevage et al. (2019)^38^	WA, USA	To conduct a mixed methods process and outcome evaluation of a F&V Rx program	Participants were eligible for program if they qualified for SNAP. Study participants recruited via convenience sampling (n = 144).	The F&V Rx included a US $10 fruit and vegetable voucher redeemable at supermarkets. No limit on the number of times a patient could receive a Rx (e.g., in some settings patients received a Rx once per week for 6 months).	Mixed methods outcome and process evaluation	Post-intervention surveys collected demographic and socioeconomic characteristics (CDC Behavioral Risk Factor tool), FS (2-item screener), and F&V consumption (Wholesome Wave F&V Consumption Survey tool). Investigators reviewed quarterly reports, meeting minutes and notes, and telephone call and email logs for process evaluation.	Documents coded to identify (1) major facilitators and barriers of program implementation; (2) key activities and/or resources for successful implementation of an incentive program in clinical setting. Rx redemption rates were calculated. Patient satisfaction was evaluated through a web-based survey.	Overall redemption rate was 54.4%. Most survey respondents (88.2%) reported eating more F&V than previously as a result of the Rx. Best practices for implementation included using the Rx to improve patient engagement and retention and to connect patients to additional services and using community networks to enhance program support and uptake.	***
Xie et al. (2021)^39^	Durham, NC, USA	To evaluate the utilization and effects of a produce Rx, including impacts on healthy food purchasing and diabetes management among participants with T2D	HCPs enrolled adults (>18 y old) who received SNAP for the produce Rx program. Study participants were recruited via purposive sampling (n = 699, including 353 with T2D).	Participants received US $40 monthly for up to 1 year redeemable for F&V at a grocery store chain	Prospective cohort study	Food transaction data collected to assess Rx utilisation. Health data from 6 months before enrollment through the end of program participation were collected from EMR, including HbA1c, BMI, and systolic BP.	Participants categorized as “frequent spenders” and “sometimes spenders.” Multivariate regressions assessed utilisation predictors and program effects on F&V purchasing, HbA1c, BMI, and BP.	Being female and older was associated with higher program utilization. Hospitalizations were negatively associated with program utilization. Frequent spender status was associated with more F&V spending (*P* < .001) and variety (*P* < .001). For participants with diabetes, there were no statistically significant relationships between program utilization and HbA1c, BMI, and systolic BP.	****
Orsega-Smith et al. (2020)^40^	DE, USA	To evaluate a fruit and vegetable Rx program offered to low-income families by pediatricians	Participants recruited by HCPs who primarily saw low-income patients (n = 41). Participants had Medicaid as primary insurance, were classified as overweight, or were a family with 2 or more children	Food boxes were distributed at participating PCFs twice per month for 1 y. The program was designed to distribute 15–25 lbs of produce to participating families at no cost.	One group pre-post measurements	Surveys measured F&V consumption, purchasing behavior, perceptions of FI, and demographic characteristics	Paired t-tests conducted to evaluate changes in F&V consumption in adults and children	Pre-post intervention vegetable consumption increased in adults (*P* < .001) and fruit consumption increased in children and adults (*P* < .05)	**
Esquivel et al. (2020)^42^	O’ahu, HI, USA	To conduct a feasibility study on a community-based pediatric F&V Rx program, including support and barriers to participation	193 Rx issued to children (2–17 years) with poor nutrition recruited by pediatricians. 33 children (from 21 parents) who used all of their vouchers recruited for interviews	Paediatricians issued Rx, which could be exchanged for vouchers redeemable for up to US $72 of fresh fruits and vegetables (US $24 per month for 3 months) at FMs	Mixed methods feasibility evaluation	Rx redemption data were collected. Semi-structured guides were used for follow-up interviews.	Descriptive statistics were used to characterize Rx redemption. Two researchers analyzed the follow-up interview responses independently, then identified themes.	Participants reported lifestyle benefits for both the child and family. Barriers to utilization included affordability and accessibility, ease of use, and child diet and/or interest in attending the FM. The evaluation identified the need to: (1) streamline referrals; (2) enhance retention; (3) quantify program impact; and (4) address barriers to participation.	***
Wetherill et al. (2018)^43^	Tulsa, OK, USA	To describe preliminary outcomes of the design and implementation of a clinic-based food pharmacy	Participants were recruited by HCPs and social workers from 2 test site clinics (n = 80)	Participants received a “food package” (including produce and shelf-stable foods), an education booklet, and recipe cards. Participants were eligible to receive another food package during clinic hours 6 additional times (once per month for 6 months).	One group pre-post measurements	Standardized surveys collected information on FS status and dietary intake. Blood pressure data were obtained through EMR.	T-tests for differences between pre- and post-intervention measures	Participants experienced significant improvement in daily dietary fiber intake (*P* < .001) and a slight yet nonsignificant increase in daily F&V intake. Mean FS did not change. Among participants who used program at least 4 times and who had high BP at enrollment (n = 17), diastolic BP significantly improved (*P* = .009).	**

BP: Blood pressure; BMI: Body mass index; CDC: Centre for Disease Control; ED: Emergency department; EMR: Electronic Medical Records; F&V: Fruits and vegetables; FM: Farmers’ markets; FI: Food insecurity; FS: Food security; HCP: Health care practitioner; MMAT: Mixed Methods Appraisal Tool; PA: Physical activity; PCF: Primary care facility; PCP: Primary care practitioner; USDA: United States Department of Agriculture; WC: Waist circumference; Rx: Prescription; T2D: Type 2 diabetes.

### Target Patient Populations

Sample sizes were generally low and ranged from 8^48^ to 883^46^ participants. The median sample size was 47.5, reflecting the large number of small-scale and pilot studies included. Interventions targeted different participant groups, including adult patients only (n = 13),^31,32,34,35,37-40,43,44,47,49,51^ pediatric patients only (n = 5),^29,30,42,46,48^ families (n = 2),^45,48^ pediatric and adult populations (n = 1),^50^ pregnant women (n = 1),^36^ and HCPs (n = 1).^33^ Eligibility of patients for food prescription interventions was generally dependent on health condition, including overweight or obesity,^29,44,46,48^ hypertension,^34,35,37,44^ diabetes,^32,44,49^ poor nutrition based on growth assessment,^42^ and high risk of chronic disease, as determined by health care clinicians.^50^ Food insecurity^30,34,35,47,50^ and low-income, as assessed by income cut-offs or eligibility for Medicaid or SNAP,^29,31,34,35,37-40,44,46,48^ were also used as inclusion criteria in several studies. Four studies did not identify any inclusion criteria based on health or socio-economic status.^41,43,45,51^ One study recruited pregnant participants from a low-income neighborhood but did not assess the socio-economic status of participants.^36^ One study did not evaluate an intervention and instead recruited health care providers to evaluate their perceptions of food prescription programs more generally.^33^

### Types of Interventions

All studies evaluated or examined food prescriptions administered in a clinical setting by a physician (including primary care providers and pediatricians) and/or allied health professional (including nurses, Registered Dietitians, nutritionists, social workers, community health workers, or midwives). Characteristics of the prescriptions varied across studies. Of the 22 food prescription interventions described in the included studies, 16 included vouchers redeemable for fruits and vegetables from farmers’ markets,^29,32,34-37,41,42,46,48,50^ mobile markets,^30,44^ and partnering supermarkets or retail chains,^31,38,39,45^ which typically included produce and healthy non-perishable foods. Four prescriptions provided access to a pre-assembled food box consisting of fresh produce^40,49^ and healthy non-perishable foods.^43,47^ One intervention provided coupons redeemable for a discount on fruits and vegetable purchased at a partnering retail store.^51^ Nine of the interventions included additional education and/or counseling activities, often administered by health care providers or trainees (e.g., medical students).^34-37,45,46,48,50,51^ Such activities aimed to improve food literacy and consumer awareness,^48,50^ increase fruit and vegetable consumption,^45,46,51^ cooking and food preparation knowledge,^46,48,50^ and nutrition goal setting.^34-37^ The duration of prescriptions and their associated incentives and activities ranged from one-time-only^30,41^ to 1 year,^39,40^ with the majority of programs operating for between 12 and 26 weeks (median 14 weeks).

### Dietary Outcomes

Impacts of food prescriptions on food affordability, accessibility, consumption, and security were evaluated using both qualitative and quantitative (i.e., survey) tools. Seven studies discussed the impacts of food prescriptions on food security,^29,31,40,41,43,45,47,49^ including five that measured pre-post intervention food security using a validated measure (including the United States Department of Agriculture [USDA] 18-item, 6-item, or an adapted 2-question household food security survey developed by Hager and colleagues (2010)^52^).^29,43,45,47,49^ Of these five studies, three reported statistically significant improvements in food security scores post-intervention,^29,45,47^ one reported an improved food security score in over half of participants but did not test for statistical significance,^49^ and one reported no change in mean food security among participants.^43^ Orsega-Smith and colleagues (2020) reported that fewer participants avoided purchasing fruits and vegetables due to their high cost following the prescription intervention,^40^ while a qualitative study on a food prescription program in Seattle reportedly improved perceived household food security.^31^

Food prescriptions were widely reported to improve the affordability^31,34-36,38,42,47,49^ and accessibility^31,39,42^ of healthy foods (and especially fruits and vegetables), thereby reducing barriers to dietary changes. A total of 13 studies reported on fruit and vegetable consumption among participants of a prescription intervention program.^31,34,35,37,38,40-43,45-47,50,51^ Of these, seven studies reported increases in fruit and/or vegetable using pre-post measurements,^38,43,50^ including four that reported statistically significant increases.^37,40,45,46^ One study reported no significant change in reported fruit and vegetable consumption.^51^ Five studies incorporating qualitative methods reported perceived increases in fruit and vegetable consumption^31,34,35,41,42,47^ but did not evaluate changes using validated survey tools. Few studies evaluated impacts of food prescriptions on dietary intake beyond fruits and vegetables, although Trapl and colleagues (2018) reported a significant decline in fast-food consumption among hypertensive patients following a 12-week food prescription program that provided weekly US $10 vouchers redeemable for fruits and vegetables at a farmers’ market.^37^ Wetherill and colleagues (2018) also reported statistically significant improvement in daily dietary fiber intake following a 1-year prescription program that included $40 vouchers per month redeemable for fruits and vegetable at a grocery store chain.^43^ In a prospective cohort study, Xie and colleagues (2021) compared “frequent spenders” and “sometimes spenders” of vouchers received through a produce prescription program and found that frequent spenders consumed a greater amount and a higher diversity of fruits and vegetables.^39^ Only one qualitative study evaluated long-term impacts on fruit and vegetable consumption after completion of a food prescription program and found that positive impacts were not maintained due to persistence of economic hardship among participants.^35^

Seven studies reported improved food literacy following food prescription programs, including knowledge related to healthy diets and dietary recommendations,^31,35,37,50,51^ comfort in attending and navigating retail environments (including farmers’ markets),^31,35-37,42,48^ exposure to a diversity of raw ingredients and dishes,^31,36,37^ and teaching cooking skills to children.^48^ Those studies that incorporated education, counseling, and/or mentorship into the prescription intervention were more likely to report improvements in food literacy among participants.

### Health Outcomes

Findings on the impacts of food prescriptions on health outcomes were mixed. Seven studies reported on at least one biometric health outcome. Two studies reported improvements in systolic^49^ and/or diastolic^43,49^ blood pressure (BP), although two other studies found no improvements in BP.^32,39^ One study reported significant pre-post reductions in body mass index (BMI) among overweight children,^45^ while another study reported a similar finding in adults.^44^ A study by York and colleagues (2020) reported that 67% of patients with type 2 diabetes receiving a prescribed weekly allotment of produce lost weight.^49^ Two other studies, however, reported no significant change in weight or BMI among adult participants.^32,39^ Three studies targeted patients with diabetes and measured pre-post intervention HbA1c (a measure of long-term glucose homeostasis)^39,49^; of these, only one reported a statistically significant decrease in mean HbA1c.^32^ Two studies evaluated pre-post physical activity (PA),^45^ of which one reported an increase in vigorous exercise among participants.^50^ One study that assessed electronic medical records (EMR) found that providing patients with prescriptions worth US $40 per month redeemable for fruits and vegetables at a grocery retailer led to lower health care utilization, including emergency department (ED) visits.^39^

Qualitative studies found that patients generally perceived food prescription interventions to be beneficial for their health, including perceived positive impacts on diet and lifestyle,^42^ chronic disease management,^38^ and nutrition or diet-related goals.^34,38^ One qualitative study reported that a food prescription program, which included US $10 vouchers redeemable at a farmers’ market, freed up household funds for other health-related costs (e.g., medication).^34^ This finding indicated that food prescription interventions may have secondary health benefits beyond those related to food access and diet.

### Referral Pathways, Patient-Provider Relationships, and Patient-Centered Care

A few studies discussed patient-provider relationships and noted the power wielded by primary care practitioners (PCPs) and allied health professionals as “authorities” and “influencers” with “expertise”, which may increase likelihood that patients will follow their recommendations and fulfill food prescriptions, in comparison to food incentive programs not linked to medical providers.^29,30,34,50^ By contrast, one intervention that included a mentorship component (run by medical students at farmers’ markets) suggested that this model leads to improved patient-centered care by reducing the inherent hierarchies in clinical medicine, rather than exploiting them.^50^ Qualitative studies with providers also underscored the potential of food prescriptions to reduce barriers to incentivize positive behavior change by giving HCPs a “ground to stand on.”^36^ In other words, food prescriptions provide a pathway for practitioners to empower participants to follow dietary recommendations by directly increasing the affordability and accessibility of healthy foods and enabling dietary changes.^33,36^ In two qualitative studies, health care providers expressed the perception that enrollment in food prescription programs increased patients’ attendance of appointments, perhaps indicating the potential of such incentives to lead to increased trust in health care systems.^36,38^

### Facilitators and Barriers to Program Utilization

Of the five studies that recorded overall redemption rates of food incentives, figures varied from 34.5%^47^ to 59%,^38,46,51^ with between 63% and 73%^39,47^ of participants redeeming vouchers at least once and between 9% and 18%^46,47^ of participants redeeming all of their vouchers. Four studies assessed the impacts and covariates of program utilization and reported that increased redemption of vouchers was associated with more fruit and vegetable spending and diversity and reduced ED visits and hospitalizations,^39^ but not food security status^29^ or fruit and vegetable consumption.^46^ Higher program utilization was associated with older age,^39^ female sex,^39^ and those who reported interest in shopping at farmers markets at baseline.^37^ Several studies cited participant retention and voucher usage as limitations of their prescription programs, and it was common for voucher redemption to decline over the duration of the intervention.^47^ A number of barriers to program utilization were identified, including transportation to food vendors,^31,34,35,48^ low food literacy and limited access to kitchen appliances,^34,48^ expiration of vouchers,^51^ limited hours and poor accessibility of farmers’ markets,^41,48^ a lack of communication with participants regarding program implementation,^41^ poor-quality produce at participating retailers,^31^ and technical difficulties and/or limited employee training regarding voucher redemption at check-out.^31,41^

Eight studies discussed facilitators of program utilization. In general, good communication between practitioners, patients, and voucher redemption locations was cited as crucial to the success of programs and encouraged utilization by participants.^33,36,42^ Robust supports and educational opportunities (e.g., through additional mentorship, nutrition counseling, and goal setting) were identified by several studies as factors that improved participants’ experiences.^33,48,50^ Some programs attempted to address transportation issues by facilitating free transportation to farmers’ markets or establishing mobile markets.^30,34^ One study mentioned patient motivation as a strong facilitator of program usage and healthy dietary change.^35^

### Study Quality and Limitations

The quality of studies is reported in Table 1 and was generally moderate or weak due to the lack of controls or comparison groups, non-randomized convenience sampling, small sample sizes, loss to follow-up and incomplete outcome data, the use of non-validated measurement tools, limited adjustment for confounders, and limited use of theoretical frameworks (for qualitative studies). Measured outcomes were variable between studies, with studies inconsistently evaluating the impacts of food prescriptions on food security, fruit and vegetable consumption, and health outcomes. Measured outcomes were often self-reported, raising questions about response bias and social desirability. Further, multi-pronged interventions (often consisting of a prescription, an incentive, and education/mentorship support) created challenges for parsing out the relative impacts of each programmatic component on outcomes of interest. The maximum duration of interventions was 1 year, although most interventions were less than 6 months, precluding the capacity of studies to determine long-term impacts of food prescription programs. Only one study evaluated post-intervention outcomes beyond 1 year after program completion.^50^

## Discussion

This scoping review identified a variety of food prescription interventions employed in primary care settings to increase access, affordability, and consumption of healthy foods, mostly fresh fruits and vegetables, among patients. The large majority of these programs have emerged in the United States, perhaps driven by the growth of Wholesome Wave (a large not-for-profit organization that operates fruit and vegetable prescription programs in 27 US states)^53^ and the US Department of Agriculture’s Gus Schumacher Nutrition Incentive Grant Program, which supports projects that incentivize fruit and vegetable purchases among participants of the long-standing SNAP.^54^ Further, in 2018, the Federal Farm Bill added a new $25 million Produce Prescription Program to implement and evaluate fruit and vegetable prescriptions in health care. It is clear that, likely in part due to these initiatives and investments, research on food prescriptions is growing in popularity, with over 85% of included studies published in the past 4 years.^54^ This rapid growth of interest in food prescriptions reflects the recent recognition of “food is medicine,” a concept and framework that encourages improved integration of healthy food supports in primary health care.^15,55^ This concept also aligns with recent calls for improved patient-centered care and the important role of health systems to address social determinants of health, perhaps establishing momentum around initiatives that leverage health care settings to address upstream determinants of health and reduce burdens on health care systems.^15^

The objectives of food prescription programs include reducing food insecurity,^45^ increasing fruit and vegetable consumption,^46^ increasing nutrition literacy,^51^ and improving health.^39^ However, evidence on the effectiveness of such programs to achieve their stated goals is limited and mixed. Overall, there is some evidence to suggest that food prescriptions can be used to improve fruit and vegetable intake^37,38,40,43,45,46,50^ and reduce food insecurity,^29,45,47^ suggesting that such programs are promising and should be investigated further. Studies incorporating health metrics reported mixed effectiveness, with little consensus on the impacts of food prescriptions on blood pressure, BMI, or glucose homeostasis.^32,39,43-45,49^ Inconsistent findings may be due in part to the poor quality and limited timeframes of studies in this nascent body of research. Most included studies were established as evaluations of small-scale (sometimes pilot) programs, rather than as well-designed research projects. As food prescription programs continue to gain popularity, it is essential that rigorous studies be undertaken to evaluate efficacy. Future research should incorporate randomization, control or comparison groups, sample size calculations to determine the appropriate number of participants, and reliable validated measures of dietary intake, food security, health, and health care utilization. For programs with multiple components (e.g., incentives, education, and mentorship), study arms should evaluate their relative efficacy to determine which components provide the greatest benefit to patients. Repeated measures should be employed to identify changes over time and follow-up with participants following termination of the intervention would identify long-term impacts. Finally, research is needed in global contexts as health care systems in other countries follow the lead of the US and implement food prescription programs.^56^

As is inherent in all “food is medicine” approaches, food prescriptions differ from other nutrition incentive programs by relying on the legitimacy of endorsement by a health care provider.^15^ In other words, food prescriptions leverage the expertise and perceived authority of health care providers to encourage patients to make dietary changes, then provide them with resources (e.g., funds/vouchers, education, and literacy) to facilitate these changes.^36^ Despite this, most reports fail to describe how the prescription is provided to the patient and no studies have investigated how the patient-practitioner relationship bears on program utilization and efficacy. While “food is medicine” approaches often endorse patient-centered care,^15^ a question that remains is whether food prescriptions contribute to patient empowerment or if they entrench (and indeed depend on) paternalistic power dynamics for which health care systems have been criticized.^57^ Future research on food prescriptions should therefore address and further explore this tension to ensure that gains in primary outcomes (e.g., fruit and vegetable consumption, food security, and health) do not come at the cost of patient empowerment.

Utilization of food incentives by participants varied across studies. Our review identified a number of facilitators and challenges of food prescription interventions, with relevance for practitioners and community organizations intending to adopt this model. Limited transportation and limited retail hours acted as barriers to accessing farmers’ markets and supermarkets, underscoring the importance of physical accessibility to participating retailers.^30,31,34-36,48^ Clear communication between practitioners, patients, and other program facilitators (e.g., food retail management and cashiers) is necessary to define clear roles and limit confusion for all program partners and participants.^33,36,41,42^ Taking steps to destigmatize program enrollment and incentives (e.g., by creating electronic voucher systems) would ensure that participants feel welcome in health care and retail environments. Further, qualitative studies reported that educational opportunities (e.g., through additional mentorship, nutrition counseling, and goal setting) improved participants’ experiences and may lead to improved program utilization.^33,48,50^ Such findings underscore how patients’ social and physical environments affect engagement, adherence, and efficacy of food prescription programs. Evaluating and understanding individual, social, and structural constraints through rigorous qualitative and mixed methods research is important to address contextual barriers that affect utilization and benefits for patients receiving food prescription.

This review was strengthened by the rigorous search process and systematic scoping review methodology. Guided by experts in academia and health care, two reviewers established the protocol a priori and conducted article screening and data extraction, which led to a robust dataset including study characteristics and findings, as well as major themes emerging from a thematic analysis. Our presentation of results and discussion contributes several important considerations for practitioners, community and health care organizations, and researchers considering implementing or evaluating food prescription programs. The review was limited by incorporating only those studies published in the academic literature, which may have excluded program evaluations published as reports by health care and community organizations.

In conclusion, food prescriptions are a promising health care-based intervention. Preliminary evidence suggests they may improve fruit and vegetable consumption and reduce food insecurity. As yet, evidence for their impacts on health outcomes is limited and mixed. Clear communication between practitioners and patients, as well as between program facilitators and food retailers (e.g., farmers’ markets and supermarkets) is necessary to ensure incentives are appropriately utilized and to destigmatize the experience for patients. Interventions should be responsive to the social and structural context within which programs are implemented to ensure strong engagement and benefits to participating patients. Addressing barriers such as stigma, accessibility challenges, transportation, and nutrition literacy may also improve patients’ experiences and increase their utilization of food prescriptions. This review has identified a clear need for further studies that incorporate larger sample sizes, control groups, and validated assessments of dietary intake, food security, and health. Such research is worth pursuing due to the preliminary successes of food prescription programs and the increasing interest in patient-centered primary health care that identifies and addresses social determinants of health, including access to nutritious foods.

## So What?

### What is Already Known About This Topic?

Food prescriptions are growing in popularity, and several recent published studies evaluate the development and impacts of food prescriptions on various health outcomes, such as dietary consumption, food security, and health.

### What Does This Article Add?

As yet, scholars have made little effort to use systematic review methods to synthesize published literature on food prescription programs. This systematic scoping review is timely and important to characterize existing evidence and identify gaps and limitations that should be addressed as food prescriptions are implemented and evaluated in national and international contexts.

### What Are the Implications for Health Promotion Practice and Research?

Our findings demonstrate that food prescriptions may improve fruit and vegetable consumption and reduce food security, although their impacts on health are uncertain. We encourage public health and health care practitionerss to implement and rigorously evaluate food prescriptions in clinical settings. Practitioners implementing food prescriptions should ensure they address barriers to use, including stigma, accessibility challenges, transportation, and nutrition literacy. Researchers should consider rigorous study designs that incorporate larger sample sizes, multiple study arms, and validated outcome assessments.

## In Brief

Food prescription programs, in which healthcare practitioners prescribe healthy foods to patients and provide them with supports to reduce barriers to accessing healthy food, are becoming increasingly popular. We conducted a systematic scoping review to identify, characterize, synthesize, and evaluate evidence on food prescription programs, including their impacts on food consumption, food security, and health. Following a systematic search and screening process, data were extracted and assessed from 23 relevant academic publications. Evidence showed that food prescriptions may improve fruit and vegetable consumption and reduce food insecurity. However, there is little consensus regarding the impacts of food prescriptions on health. The quality of included studies was weak, underscoring a need for rigorous research that incorporate larger sample sizes, control groups, and validated assessments. In sum, food prescriptions are a promising health care intervention that warrant further investigation.
